# Variability of chlorophyll-a concentration in the Gulf of Guinea and its relation to physical oceanographic variables

**DOI:** 10.1016/j.pocean.2016.11.009

**Published:** 2017-02

**Authors:** Karen Nieto, Frédéric Mélin

**Affiliations:** European Commission, Joint Research Centre, Via E. Fermi, 2749, 21027 Ispra, VA, Italy

**Keywords:** The Gulf of Guinea, Oceanography, Remote sensing, Chlorophyll, Spatial variations, Physical variables, Mesoscale, Fronts, Eddies

## Abstract

The Gulf of Guinea represents a wide tract of the African coast with complex and rich coastal ecosystems undergoing various pressures. The seasonal variations of chlorophyll-a concentration (Chla) along the Gulf of Guinea (GoG) and their relations with physical oceanographic variables were analyzed using satellite observations covering the period 2002–2012. The effects of sea surface temperature (SST), sea level anomalies (SLA), winds, geostrophic currents, eddy kinetic energy (EKE), mesoscale eddies and fronts were considered on a monthly time scale. The analysis for each unit area was carried out on a chlorophyll index (IChla) computed as the product of the mean distance from the coast to the eutrophic threshold (1 mg m^−3^ isoline) and the average Chla in the eutrophic area. The study, based on satellite-derived Chla, was allowed by the unprecedented coverage given by the products distributed by the ESA Ocean Colour Climate Change Initiative (OC_CCI) resulting from the merging of data from several satellite missions. The physical variables served as potential predictors in a statistical Boosted Regression Tree (BRT) model. To account for the heterogeneous nature of the GoG, the analysis was conducted on eight systems that made up a partition of the whole region defined on the basis of the BRT model results and climatological properties. The western-most domain, from Guinea-Bissau to Sierra Leone, was associated with upwelling properties in boreal winter and appeared to share some characteristics with the overall Northwest African upwelling system. The region of Ivory Coast and Ghana also had upwelling properties but the main upwelling season was in boreal summer. In general upwelling conditions with cold SST, negative SLA, fairly strong frontal activity, and moderate winds, appeared as the environmental window most favorable to high IChla values. For these systems, the BRT model fitted the IChla data well with a percentage of explained total deviance $D^{2}$ between 70% and 91% when using only physical oceanographic variables. Finally, the systems associated with the coasts of Nigeria to Gabon showed some mixed properties, with $D^{2}$ values of 54–60%. Among these systems, a common feature seemed to be the importance of river discharge to explain IChla variations. Where possible (for the Niger River in the Nigeria system), the addition of river data as predictor in the BRT model resulted in a significant increase of $D^{2}$ to 75%. Further progress is needed to understand the observed relationships and to predict how they can evolve in the face of climate change.

## Highlights

• Satellite data were used to characterize the oceanography of the Gulf of Guinea.
• Ocean color CCI Chla offered unprecedented data coverage of the Gulf of Guinea.
• Chla was modeled as a function of SST, SLA, winds, currents, EKE, eddies and fronts.
• Chla was higher in upwelling conditions with cold SST, negative SLA, strong fronts.
• The impact of river outflow on Chla was significant mostly from Nigeria to Gabon.

## 1 Introduction

**Fig. 1:**
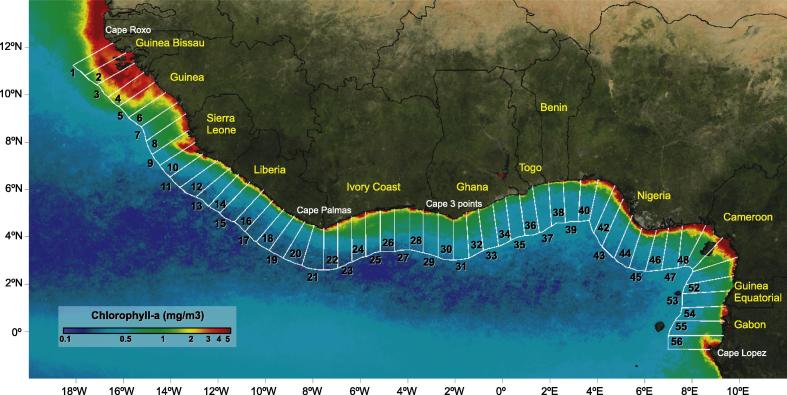
Study Area: Partition of the domain into cells (from 1 to 56) over-imposed to Chla mean distribution from 2002–2012 (CCI data).

The Gulf of Guinea is a dynamic and complex ecosystem along the equatorial West African coasts, approximately extending from Guinea–Bissau to Gabon (Fig. 1). Located near the equator, this tropical region is characterized by warm waters and abundant light that promotes the growth of plankton which in turn supply food for fishes and sustain fisheries, a key source of revenue for economic and social development for the countries of the region.

According to *Sea Around Us* information (Pauly and Zeller, 2015), in the Guinea Current Large Marine Ecosystem (GCLME) which encompasses the Gulf of Guinea (Sherman and Duda, 1999), the pelagic fishes, such as sardines, anchovies, herring and tunas, were fished by eighty-four countries during 2000–2010 (see also Belhabib et al., 2015), with pelagic catches accounting for 34–38% of the total catches in the area. Ghana has historically been the major fishing country in the region, but the activity has been affected by variations in pelagic catches (Atta-Mills et al., 2004, Nunoo et al., 2014, Pauly and Zeller, 2015). Ghana, Nigeria and Sierra Leone were the major fishing countries in the Guinea Current LME during 2000–2010 with 31%, 16% and 14% of the pelagic catches (Pauly and Zeller, 2015).

In addition to pressures of anthropogenic origin (Ukwe et al., 2003), such as those associated with fishing practices (Kaczynski and Fluharty, 2002, Atta-Mills et al., 2004, Stewart et al., 2010) and various sources of pollution, the Gulf of Guinea is facing climatic and oceanographic variability affecting the distribution and abundance of phytoplankton and therefore the whole pelagic food web. Oceanographic conditions dominating the region are the Guinea Current and Undercurrent, coastal upwelling, fronts, mesoscale eddies and the influence of rivers, lagoons and mangroves. Most oceanographic studies of the Gulf of Guinea have focused on physical variability (Hardman-Mountford and McGlade, 2002, Djakoure et al., 2014) while a few investigations have focused on phytoplankton; most analyzes have been carried out using *in situ* or satellite data in a specific season and area (Djagoua et al., 2011), even though there are some exceptions (e.g., Bakun, 1978, Binet, 1983). To the best of our knowledge, no previous study has used observations to develop quantitative models on the effects of physical variables on the distribution of the chlorophyll-a concentration (Chla) at the scale of the entire region.

An obstacle to study the Gulf of Guinea using satellite remote sensing has been the scarce availability of sea surface chlorophyll and temperature data due to atmospheric contamination (Hardman-Mountford and McGlade, 2002, Kouadio et al., 2013, Estival et al., 2013). This tropical region, close to the Inter-Tropical Convergence Zone (ITCZ) benefits from a high level of incoming solar radiation, which is released mainly through evaporation, contributing to high cloud coverage in the area. Variables such as ocean color and sea surface temperature (SST) cannot be measured in those conditions by visible and infrared remote sensing. Aerosols from dust or biomass burning (Resch et al., 2008, Real et al., 2010) contribute further to the challenges facing the process of atmospheric correction of remote sensing data. As a consequence, most single days in this region offer very few useful ocean-color data, and complete maps of ocean-color products can only be achieved using composite images over weeks to months. The permanence of clouds or aerosols in some periods means that even multi-annual monthly climatological maps may show gaps (which is to say no valid data) over part of the domain (Djagoua et al., 2011, Kouadio et al., 2013). The products from the European Space Agency (ESA) Ocean Colour Climate Change Initiative (OC_CCI) used in this work, resulting from the merging of data from several satellite missions, were conceived to improve the spatial coverage with respect to standard single-mission products in regions affected by clouds and aerosols, which greatly supports the objectives of this study.

The main objective of the study was to revisit the seasonal variations of Chla within the coastal regions of the Gulf of Guinea benefiting from the upgraded spatial coverage afforded by the OC_CCI products, and to relate these variations with physical oceanographic variables. Previous studies that divided the Gulf of Guinea into subsystems to account for the heterogenous nature of its oceanography based the division mostly on expert judgment, and not on a quantitative approach. A first attempt in that direction was made by Hardman-Mountford and McGlade (2002) using only satellite SST data to define subsystems. Here the partition of the Gulf of Guinea was achieved by adopting a single modeling framework for the entire domain linking Chla with a comprehensive set of physical variables. The final aim of the study was to shed some light on the mechanisms driving the variations in phytoplankton, as represented by chlorophyll-a concentration, in the various coastal regions along the Gulf of Guinea.

## 2 Data and methods

This section presents the various data sets prepared for the study before introducing the methods of analysis. The domain was partitioned into 56 individual cells covering the coast from Cape Roxo in Guinea–Bissau (12°20′N, 16°45′W) to Cape Lopez in Gabon (0°38′S, 8°42′E) (Fig. 1). It is acknowledged that some authors defined slightly different boundaries for the Gulf of Guinea (Longhurst, 1962, Binet and Marchal, 1993, Djagoua et al., 2011), for instance selecting the Bissagos Islands (Guinea–Bissau) just south of Cape Roxo as the northern limit. Each cell has a length (from coast to offshore) of 200-km (covering a large part of the exclusive economic zone and the whole local shelf), and a width equal to 1/3 of the length (∼67 km) to guarantee a sufficient discretization of the coastline while maintaining a manageable number of cells. The cell was the basic unit area for which statistical analysis was conducted.

### 2.1 Chlorophyll-a concentration

We used the sea-surface chlorophyll-a concentration data from the Ocean Colour CCI (OC_CCI, Version 1, CCI, 2011) project, a merged product combining data from the US National Aeronautics and Space Agency (NASA) Sea-viewing Wide Field-of-view Sensor (SeaWiFS, McClain et al., 1998) and Moderate Resolution Imaging Spectroradiometer (MODIS, Esaias et al., 1998) onboard the Aqua platform, and from ESA’s Medium Resolution Imaging Spectrometer (MERIS, Rast et al., 1999). After the process of atmospheric correction, Chla was computed by the maximum-band-ratio algorithm OC4v6 (O’Reilly et al., 2000). The coverage of the OC_CCI merged Chla images was compared with that provided by the single-mission products (SeaWiFS, MODIS and MERIS) obtained from NASA and processed following the same algorithms (Franz et al., 2007), and with the coverage given by a simple merging of these three products. Only part of the increase in coverage (see results in Section 3.1) afforded by OC_CCI resulted from the merging of multiple missions. An additional gain in valid data was provided by processing the MERIS imagery with the atmospheric correction *Polymer* (Steinmetz et al., 2011) instead of the standard atmospheric correction used by NASA. The main advantage of *Polymer* is that it was designed to provide valid ocean color data in presence of sun glint (specular reflection of the sun rays on the water surface); additionally, it showed a good robustness to the presence of thin clouds and aerosols (Müller et al., 2015). In the OC_CCI processing, this was coupled with a better discrimination of clouds leading to a high quality cloud mask for the atmospheric correction (CCI, 2011).

The period of analysis covered the interval 2002–2012, when the OC_CCI merged product reached its maximum coverage with the addition of the MERIS and MODIS data records to SeaWiFS (from April 2002 and from July 2002, respectively). The level-3 data were available on a daily basis (amounting to 3865 daily images) and with a 24th-degree grid (approximately 4.6 km spatial resolution) and were obtained from http://www.esa-oceancolour-cci.org. Despite the improvement in coverage, daily data were still affected by the cloud cover associated with the region, which explains why monthly composites were used to analyze the relations between Chla and physical oceanographic variables.

Considering the focus on coastal regions, a chlorophyll-a concentration of 1 mg m^−3^ was used as a threshold to identify eutrophic coastal waters. This threshold has been used to compare primary production in different systems (Nieto, 2009). The analysis aimed at illustrating the links between physical variables and Chla offshore of any location along the coast of the domain (as represented by the cells shown in Fig. 1). Conditions favorable to algal growth can be translated into a local increase in Chla as well as an extension offshore of the high-biomass region. To accommodate both manifestations, the quantity selected as variable for the analysis in each cell was the product of the mean distance from the coast to the eutrophic threshold (1 mg m^−3^) and the average Chla in the eutrophic area. This product, termed the Chla index (IChla), represented the integrated areal Chla (in mg m^−2^) within the eutrophic part of each cell. It can be noted that average Chla and distance to the 1 mg m^−3^ isoline were often well correlated, even though there were exceptions (Appendix 2). The Chla and IChla fields were log-transformed (base 10) prior to analysis, consistently with a hypothesis of log-normal distribution for these variables.

### 2.2 Satellite sea surface temperature

The Microwave (MW) plus Infrared (IR) Optimally Interpolated (OI) SST is a merged product that combines the capabilities of free-cloud observation of MW data and the high spatial resolution of IR data. The Microwave plus Infrared (MW_IR) OI SST Level 3 data used in this study were created by Remote Sensing Systems (http://www.remss.com/). Microwave SST data came from the Tropical Rainfall Measuring Mission (TRMM) Microwave Imager (TMI), the Advanced Microwave Scanning Radiometer (AMSR-E and AMSRT2) and WindSat satellite missions. Infrared data came from MODIS on-board both Terra and Aqua satellites. The Level 3 SST product had a spatial resolution of 1/12° (approximately 9-km) on a daily basis and was available from June 2002 to present. Images were resized by expanding the number of columns and rows (without interpolation) in order to match the grid associated with the Chla data (1/24° resolution). A similar treatment was performed for the other physical variables to allow the analysis on a common grid.

### 2.3 Sea level anomalies and geostrophic currents

Sea Level Anomalies (SLA) and Geostrophic Currents (GC) from satellite altimetry produced by Ssalto/Duacs were obtained from Archiving, Validation, and Interpretation of Satellite Oceanographic data (AVISO), with support from the French Centre National d’Etudes Spatiales (http://www.aviso.oceanobs.com/duacs/). A recent processing carried out by AVISO allowed us to use daily altimetry data (SSALTO/DUACS, 2015). The satellite altimetry data were generated from a merged product of TOPEX/Poseidon, Jason-1, ERS-1 and Envisat satellites, with the computation of SLA based on a reference period of 20 years (1993–2012). The zonal (*U*) and meridional (*V*) components of the GC daily products (computed by AVISO from SLA at a 0.25° resolution) were used to compute the Eddy Kinetic Energy (EKE) associated with the geostrophic flow (EKE = $(U^{2}+V^{2})/2$). The GC were also used to detect eddies (see Section 2.5). For the current analysis, monthly products of SLA and EKE were derived from the daily fields.

### 2.4 Ocean winds

Daily level-4 gridded mean wind fields from satellite scatterometer data were produced and distributed by CERSAT-Ifremer (http://cersat.ifremer.fr/). We used the NASA SeaWinds scatterometer data, onboard the QuikSCAT satellite (0.5° resolution) for the period 2002–2008 and the ASCAT scatterometer data, onboard the Metop platform (0.25° resolution) for the period 2009–2012. Monthly images of wind speed and direction were then created from the daily data.

### 2.5 Mesoscales structures: fronts and eddies

Indicators related to the occurrence of fronts and eddies were included as explanatory variables considering their role in marine ecosystems (e.g., Bakun, 2006, Nieto, 2009, Nieto et al., 2014). The MW_IR OI SST, henceforth SST data, were used to detect SST fronts using the Sobel edge operator (Sobel and Feldman, 1968). This gradient approximation is based on a 3 × 3 convolution mask and the result for each pixel in an image is the corresponding gradient vector. Gradient distributions were first computed using monthly SST maps; the centroid of these distributions was then determined within each cell to locate the main frontal region. Finally a front index was computed as the product of the average gradient in the cell by the distance from the coast to the centroid (the front index was therefore expressed in °C, as the product between a temperature gradient (°C km^−1^) and the gradient centroid distance in km). This lessened the importance of the frontal structures existing very near the coast while giving more weight to offshore structures often associated with the extension of Chla patterns.

Mesoscale eddies were detected using both components of geostrophic currents from daily data and following the methodology described by Nieto et al. (2014). The current fields were first interpolated onto the 1/24° grid of the ocean color data. The eddy detection relied on the calculation of the Okubo-Weiss (OW) parameter (Okubo, 1970, Weiss, 1991). The core contours of eddies were defined as areas where the OW parameter was less than −5e^−11^ and confirmed by visual inspection of images depicting geostrophic currents in the study area. A global threshold commonly used in published literature (-2e^−12^) overestimated the number of eddies while the lower adopted threshold allowed a better eddy identification for the specific study area (Nieto et al., 2014). The rotational sense of the eddies (cyclonic or anticyclonic) was determined by computing the direction of the velocity current vectors within the eddy. Each grid point of each daily map was associated with a cyclonic eddy/anticylonic eddy/non-eddy binary diagnostic according to the value of the OW parameter at that point. The presence of eddies for each month and cell was then obtained by taking the maximum number of eddy occurrences (in days) within the cell during that month. This effectively quantified the presence of eddies affecting the cell while accounting for the fact that eddies were possibly moving from one cell to another.

### 2.6 River inputs

**Fig. 2:**
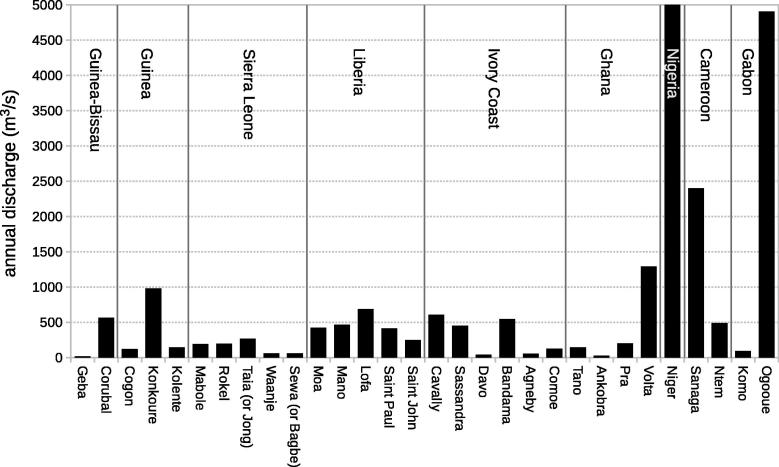
Annual river discharge from rivers feeding the Gulf of Guinea.

Another factor included in the study was the influence of river discharge, which has been proposed as a local mechanism to explain chlorophyll-*a* maximum over the continental shelf in some coastal regions of Western Africa (Kouadio et al., 2013) as nutrients and organic matter enter the marine ecosystems (da Cunha and Buitenhuis, 2013, Arauja et al., 2014). In order to identify the most relevant rivers, annual averages of water discharge associated with rivers feeding the GoG were compiled from the SIEREM (Système d’Informations Environnementales sur les Resources en Eau et leur Modélisation, http://www.hydrosciences.fr/sierem) (Fig. 2).

Among the various river courses feeding the Gulf of Guinea, the Niger River has the largest flow (Fig. 2) and its delta ecosystem is composed of numerous estuaries, rivers, creeks, lacustrine and inshore waters which support freshwater and brackish mangrove swamps. Nigeria has the largest area of mangrove forests in Africa (UNEP, 2007). A hypothesis was considered postulating that the high values of Chla found in the Nigeria system were affected by the input of nutrients and organic matter associated with the Niger River. At Lokoja station in Nigeria, the average annual discharge for the period 1971–2001 was 5066 m^3^ s^−1^ (Abrate et al., 2013).

This hypothesis was tested using satellite hydrology data, downloaded from the Laboratoire d’Etudes en Géophysique et Océanographie Spatiales (LEGOS) web site (http://www.legos.obs-mip.fr/soa/hydrologie/hydroweb/Page_2.html). The database contained time series of water level for large rivers around the world. These series, based on altimetry data from Topex/Poseidon, ERS, Envisat and Jason-1 satellites, corresponded to fixed locations (according to satellite orbits) equivalent to virtual hydrological stations. To study the links with variations offshore, time series of the most downstream stations along the Niger River were considered over the period November 2002 to July 2010 with data from the Envisat satellite. Based on Envisat’s repeat orbital cycle, the temporal resolution of the water level time series was 35 days. The stations were located in the delta with the following position: 5°59′N – 6°43′E and 6°39′N – 6°39′E. In order to have a representative monthly value of water level to compare with the monthly IChla, the water level on the 15th day of each month was computed by linear interpolation of the values associated with the nearest dates.

Another river of regional importance was considered, the Ogooue river in Gabon, with an average annual discharge of around 3800 m^3^ s^−1^ over the period 2000–2007. The monthly data published by Mahé et al. (2013) were compared with the annual cycle of IChla in the system including the coast of Gabon (note that these discharge data may differ from those compiled by the SIEREM and shown in Fig. 2 because of different dates and sources). The Sanaga river in Cameroon is also an important river with an average annual discharge of 1720 m^3^ s^−1^ over the period 1997–2004. The monthly discharge data at Songmbengue station in Cameroon for the period 1997–2004 published by Siddi et al. (2014) were used to compute a monthly average climatology, which was compared with the annual cycle of IChla in the Cameroon system.

The other rivers feeding the GoG generally appeared to have a relatively small flow (Fig. 2), which was consistent with the study of Djagoua et al. (2011) showing that the presence of turbid waters was not significant from Sierra Leone to western Nigeria. Specific cases of secondary rivers are mentioned below only when appropriate. In all cases except the Niger River, the scarcity of river discharge data with the appropriate temporal resolution limited their full inclusion in any statistical modeling.

### 2.7 Data analysis

The relationship between the chlorophyll index IChla, taken as the dependent variable, and physical oceanographic variables was explored using a Boosted Regression Tree (BRT) analysis. A strength of BRT modeling is that it can fit complex nonlinear relationships and automatically handle outliers and interaction effects between predictors (Elith et al., 2008).

An initial set of BRT models was derived at the level of individual cells to account for the heterogeneity of the GoG and in order to avoid an arbitrary partition of the region. A total of 132 monthly data (11 years) for each cell was available for the study period considering data from 2002 to 2012. The BRT construction was performed using the “dismo” package (Elith and Leathwick, 2016) in R version 3.1.1 (R Development Core Team, 2014). The selection of the settings for the BRT model followed the guide of the R “dismo” package, with a reasonable starting point to run the model defined by tree.complexity  = 5, learning.rate  = 0.01 and bag.fraction  = 0.5 (Elith and Leathwick, 2016). Elith and Leathwick (2016) recommended fitting models with at least 1000 trees, for which the learning.rate was adjusted in order to aim for over 1000 trees and get the definitive BRT model. In this study the adjusted learning.rate was between 0.01 and 0.05.

**Fig. 3:**
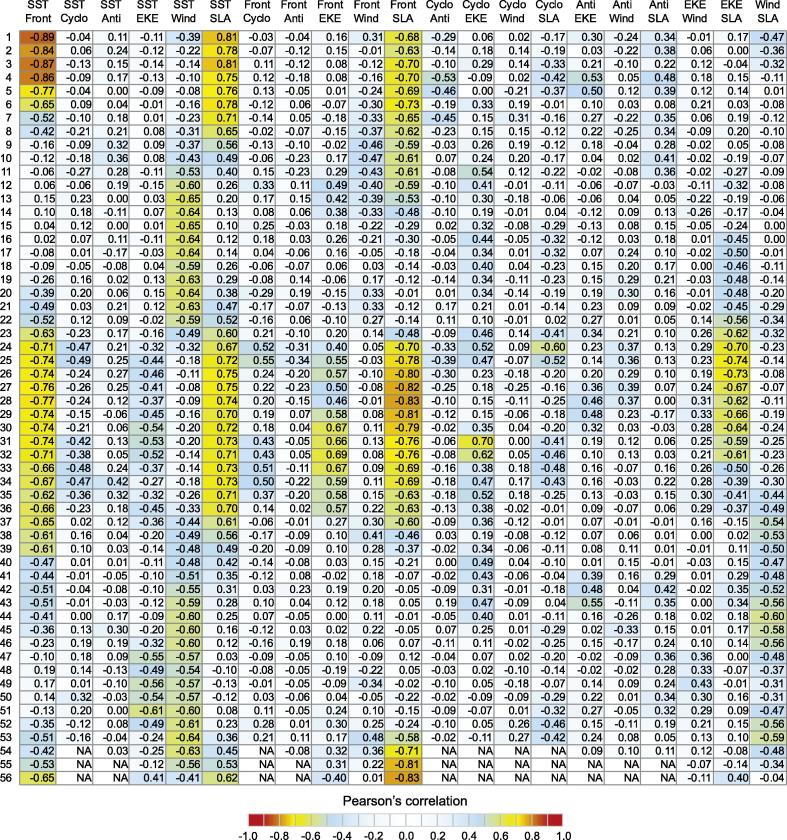
Pearson’s correlation coefficient between physical variables for each cell. “Cyclo” and “Anti” indicate the persistence of cyclonic and anti-cyclonic eddies, respectively,“Wind” is for wind speed, and “Front” for front index.

To simplify the model and get the definitive BRT model, the existence of redundant and negligible predictors was investigated. The redundancy of predictors was examined using the Pearson’s correlation coefficients (*r*) between predictors for each cell (Fig. 3), a high correlation linking a pair of predictors meaning that one predictor variable could be excluded. The threshold of absolute values of *r* to be considered as high correlation varies between BRT applications. Values of *r* greater than 0.9 (in modulus) were considered as high correlation. Unimportant predictors were those with a relative influence lower than 1%, which were not included in the final BRT model. However, since no predictors fitted these criteria (|r|>0.9 or relative influence <1%), all the predictors were kept in the definition of the definitive BRT model, which offered the advantage of having the same set of descriptors across the whole GoG.

To quantify how well the BRT model fitted the data, the percentage of deviance explained was used. This pseudo determination coefficient, $D^{2}$ (Mateo and Hanselman, 2014), was calculated with the formula:

(1) $$D^{2}=1-(\mathrm{residual}\;\mathrm{deviance}/\mathrm{total}\;\mathrm{deviance})$$

The physical oceanographic variables used as potential predictors were SST, SLA, EKE, wind speed and direction, front index and number of eddies (cyclonic and anti-cyclonic). The variable time (i.e., month) was not included as potential predictor, assuming that seasonality was already well reflected in the annual cycles associated with physical variables. Nor was the river discharge considered as default explanatory variable: in the framework of the BRT modeling, the emphasis was on conducting the analysis with the same set of variables for all cells to ensure a consistent comparison across the GoG, while river discharge is a local phenomenon. The inclusion of river data in the BRT modeling anyway was hampered in most cases by the absence of data for all cells at a monthly time scale over the full period of study. An exception was the Niger Delta discharge, for which a monthly data set was available (see Section 2.6), which allowed the inclusion of this variable in the BRT modeling performed for the cells located along the coast of Nigeria.

Based on the result of the BRT model, the relative influence of each predictor on IChla could be analyzed. The percentage of relative influence quantified the importance of predictors and its measure was calculated by the contribution of each term in reducing overall BRT model deviance (Elith et al., 2008). The relative influence was scaled so that the sum added to 100% (high relative influence values corresponding to the strongest influence on the response). The partial dependency plots resulting from the BRT analysis were also presented in order to analyze the effect of variations in the predictors on the response (i.e., on IChla) for each system.

The relative influence of predictor variables was used to characterize each cell in order to identify ensembles of contiguous cells with similar patterns of variability and to define a partition of the GoG into appropriate systems. Once this division was defined, a BRT analysis was run again for each system in order to interpret the influence of the physical variables in the area. This data reduction allowed the presentation of the results for each system instead of for all cells. To further characterize the local systems, the results of the BRT analysis were completed by climatological Hovmöller diagrams that illustrated the annual cycles of the main variables along the entire domain. It is stressed that, even though the emphasis of the study was on seasonal variations, the BRT analysis was actually conducted on the monthly data over the full period.

## 3 Results

### 3.1 Satellite data coverage

**Fig. 4:**
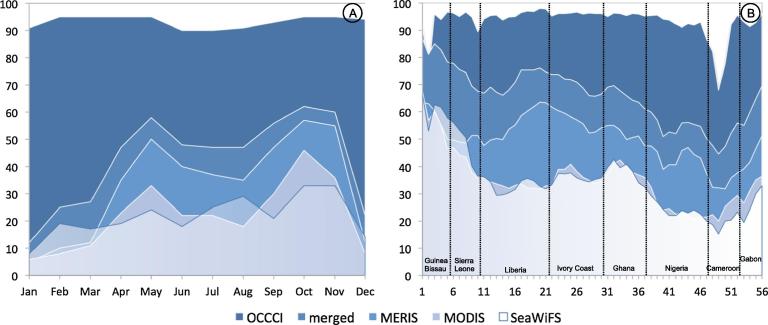
Chla valid pixels for the single mission and merged products (mean 2002–2012): (A) Percentage by month (0–200 km) for all cells, (B) percentage by cell over the year. The “merged” product is a simple combination of the SeaWiFS, MERIS and MODIS products.

The coverage of the Chla product used in this work was a great improvement to study the biological oceanography of the region: the OC_CCI monthly data covered on average 93% of the coastal domain (from the coast to 200 km offshore) (Fig. 4), which represented a major gain when compared to the average obtained using single missions: 33%, 22% and 21% for MERIS, MODIS and SeaWiFS respectively, with maxima barely exceeding 50%. Another important result was that the spatial and temporal variations in the percentage of coverage were minimal for the OC_CCI product. In contrast, for the single missions, a spatial and temporal variability was observed: geographically, the coverage associated with single mission products was found lowest in the eastern part of the Gulf (Nigeria-Cameroon, Fig. 4B), while temporally a distinct minimum in winter (December-January) was observed on average in the GoG (Fig. 4A). A simple combination of the three NASA products (named “merged”) increased the coverage to an average of 43% of the domain area, still much lower than the OC_CCI data coverage (Fig. 4), a result anticipated in Section 2.1. This enhanced data availability, well distributed across the seasonal cycle, opened some unprecedented opportunities of analysis compared with previous data sets for which even a multi-annual climatology showed missing data in winter (e.g., Djagoua et al., 2011).

### 3.2 Partition of the domain

**Fig. 5:**
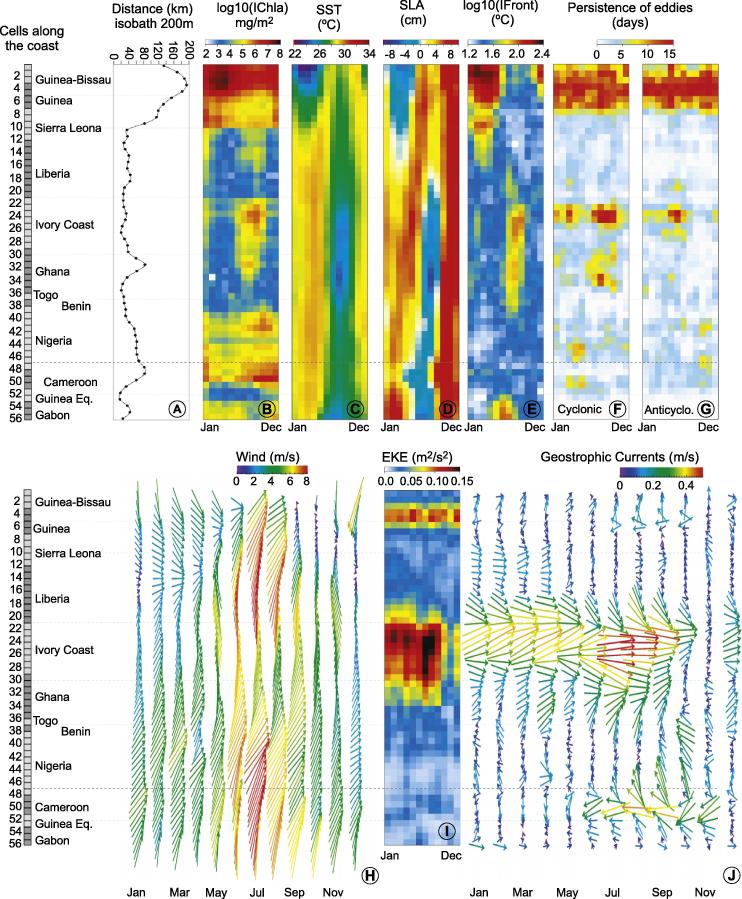
Climatological Hovmöller diagrams by cell division (1–56): (A) Distance to isobath 200-m, (B) log_10_ of IChla, (C) SST, (D) SLA, (E) Front Index, (F) Cyclonic Eddies, (G) Anticyclonic Eddies, (H) Wind: Magnitude and Direction, (I) EKE, (J) Geostrophic Current: Magnitude and Direction. The countries are indicated along the cell axis. Dotted lines show the partition into systems (as per Table 1).

The annual cycles of Chla and explanatory physical variables are shown for the domain in Fig. 5. Spatially the highest values of Chla and its extension offshore were well correlated with the continental shelf width, represented here by the 200-m isobath (Fig. 5A; the correlation coefficient between the off-shore positions of the 200-m isobath and the 1 mg m^−3^ isoline was found equal to 0.90). This relationship is observed in most coastal upwelling systems (Nieto, 2009) where narrow continental shelves contribute to a reduced extension of Chla offshore. For the region of study, this correlation was driven mostly by the high values found in the western part of the domain in association with the widest shelf; it was less clear in some other parts of the GoG, for instance in Ivory Coast where high values of Chla were not associated with a widening of the shelf. A positive correlation was also observed between the continental shelf width and offshore mesoscale patterns such as fronts and both cyclonic and anticyclonic eddies (Fig. 5A, E, F, and G). Again this relation was mostly the result of the high values found in the western part of the domain, while it was not clear for the rest of the GoG. As shown by Nieto et al. (2014) for the California Upwelling System, in the coastal areas both cyclonic and anticyclonic eddies play an important role to entrain productive shelf waters offshore. Most of the eddies were concentrated in the northern part of the system (Guinea–Bissau - Guinea), to a lesser extent along the coasts of Ivory Coast and Ghana, and in some specific cells in Nigeria and Cameroon (Fig. 5F and G).

**Fig. 6:**
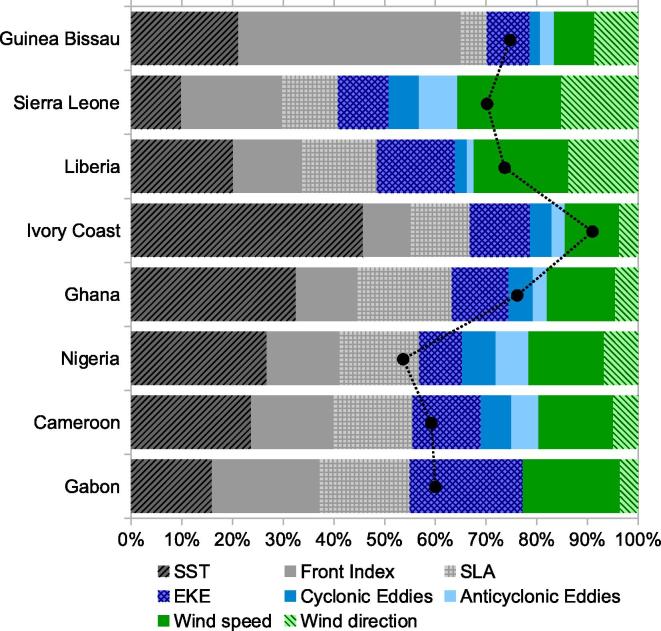
Percentage of deviance explained $D^{2}$ (black dashed line) and relative influence of physical variables on IChla and for each system.

From Appendix 1, it was seen that the contribution of the explanatory variables in the BRT model varied largely along the domain, highlighting the heterogeneity of the relations between IChla and physical quantities. Based on the results of the BRT model for each cell (Appendix 1) and the Hovmöller diagrams (Fig. 5), eight systems were defined (see numbering of the cells in Fig. 1) and named according to the main riparian countries: (i) Guinea–Bissau (cells 1–5), (ii) Sierra Leone (cells 6–10), (iii) Liberia (cells 11–21), (iv) Ivory Coast (cells 22–30), (v) Ghana (cells 31–37), (vi) Nigeria (cells 38–47), (vii) Cameroon (cells 48–52) and (viii) Gabon (cells 53–56). The vertices (longitude, latitude) for each system are listed in Table 1. When applied to these systems the BRT model accounted for a mean total deviance *D*^2^ of 54% to 91%, with the contribution of each explanatory variable displayed in Fig. 6 (river discharge excluded). $D^{2}$ values sorted from high to low were: Ivory Coast (91%), Ghana (76%), Guinea–Bissau (75%), Liberia (74%), Sierra Leone (70%), Gabon (60%), Cameroon (59%) and Nigeria (53%). Detailed results for each system are described in the next sections. Generally it was observed that the BRT model explained more variance for the systems associated with clear upwelling characteristics such as Ivory Coast, Ghana and Guinea–Bissau. To the contrary, the lowest $D^{2}$ was obtained for the Nigeria system, where it was suspected that freshwater discharge could play an important role in Chla variations. This role was not included in the model when applied to the whole Nigeria system but it can be seen at the local (cell) level in Appendix 1. The case of river discharge will be further discussed when describing regional results.

### 3.3 Partial dependency plots

**Fig. 7:**
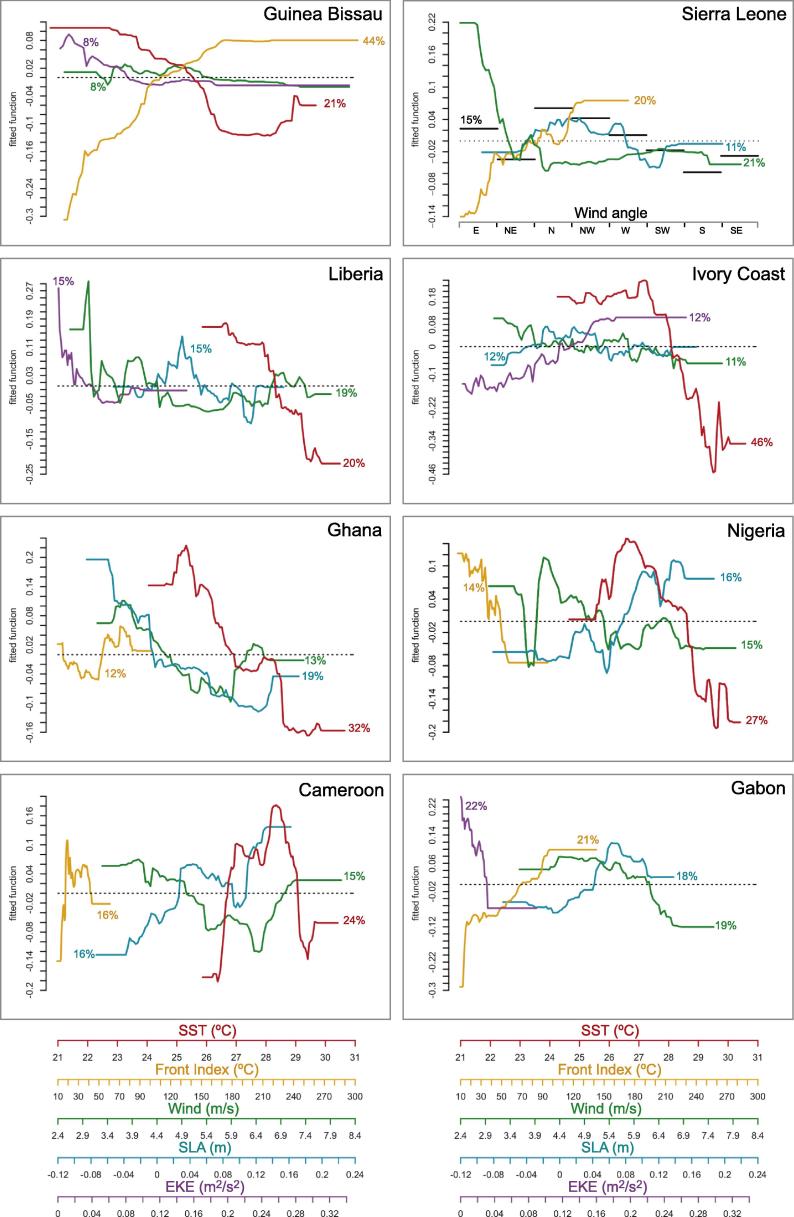
Partial dependency plots for the four most influential variables in the BRT model for each system. Fitted functions (*Y* axes) are centered by subtracting their mean. The percentage associated with each curve is the relative influence of the variable (see Fig. 6). In the case of the Sierra Leone system, wind direction is shown in black with a specific axis showing directions.

The visual representation of the effect of predictors on the response are shown in Fig. 7, describing how IChla varies as a function of the adopted predictors. The ordinate axis represents centered fitted functions of IChla, with the average IChla associated with the 0 value along the *Y*-axis (or equivalently the dotted *X*-axis). For each system, only the four most significant physical variables, in terms of relative influence on IChla, were plotted. In general, the most significant predictors were SST, Front Index, Wind speed, SLA and EKE with average relative influence for all systems of 24%, 19%, 15%, 14% and 13%, respectively (Fig. 6). The variables Wind direction, Cyclonic and Anticyclonic eddies had a lower relative influence, on average 8%, 4% and 4%, respectively.

SST had the highest relative influence for five of the eight systems (sorted from high to low): Ivory Coast (46%), Ghana (32%), Nigeria (27%), Cameroon (24%) and Liberia (20%). It was also the second most important predictor in the Guinea–Bissau system (21%). In the systems of Sierra Leone and Gabon, SST was not among the four most significant variables. In general, relatively cold SST had a positive influence on IChla, with the systems of Guinea–Bissau, Liberia and Ivory Coast having a similar relationship between SST and IChla: IChla was below its average above a threshold of 25.5 °C, 28.3 °C and 28.1 °C, respectively, while a decrease in SST had a positive effect on IChla until a plateau was reached (at 22.7 °C, 26.6 °C and 27.2 °C, respectively) in association with the maximum of IChla. A similar relationship was observed for the Ghana and Nigeria systems: below a threshold of 26.9 °C and 28.6 °C, respectively, IChla was above its mean and generally IChla increased as SST decreased. But after reaching an optimum SST (25.3 °C and 26.6 °C, respectively), IChla decreased for colder SST even though it remained above the mean. For the Cameroon system, an optimal range between 26.7 °C and 29.1 °C was observed, with the maximum positive effect on IChla at 28.3 °C; outside this range IChla decreased sharply.

The Front Index (in °C, see Section 2.5) was identified among the four most important predictors in six of the eight systems. This variable was the most important predictor in the Guinea–Bissau system with a relative influence of 42% on the statistical modeling of IChla. For the other systems, the Front Index had a relative influence of 12–21% (sorted from high to low): Gabon (21%), Sierra Leone (20%), Cameroon (16%), Nigeria (14%) and Ghana (12%). Generally, IChla tended to be high for high values of the Front Index. This was particularly clear for the three systems with the highest relative influence from the Front Index, Guinea-Bissau, Sierra Leone and Gabon: IChla regularly increased with the Front Index, reaching the average value for a Front Index of 110 °C, 76 °C and 70 °C, respectively, and a plateau at 172 °C, 130 °C and 97 °C, respectively. In the Ghana system, IChla was larger than its average value for a Front Index of 53 °C, but there was no clear relationship above that. An optimal range of Front Index between 17.3 °C and 41.5 °C was observed in the Cameroon system. In contrast, a negative relationship between Front Index and IChla was observed for the Nigeria system.

Wind speed was identified among the four most important predictors for all systems. The dependence on wind speed was made more complex by the fact that for a given amplitude, the effect on IChla might vary with the wind direction. However wind direction was listed among the four most important variables only in the case of Sierra Leone (relative influence of 15%), where wind speed happened to have the highest relative influence (21%). Wind speed was the second most important predictor for the Liberia system (19%), an area where wind direction also achieved a relatively high relative influence (14%, Fig. 6). In general, lower to moderate winds seemed to have a positive influence on IChla, while high wind speeds were often associated with low IChla. A secondary optimal range could be seen in some systems (Liberia, Ghana, Nigeria). The Guinea–Bissau system showed an optimal range for wind speeds between 3.5 and 5.5 m s^−1^. IChla values above average in association with the lowest speeds (below 3.3 m s^−1^) were an infrequent occurrence found mainly in November in some specific years (2006, 2010 and 2011), while the climatological maximum of IChla happened in February-April (Fig. 5). In the Sierra Leone system, there was a clear negative relationship with IChla for wind speeds between 2.5 and 4 m s^−1^; these moderate speeds appeared particularly effective in association with upwelling-favorable directions from the northwest. IChla in the Liberia area was usually above average for wind speeds below 4.2 m s^−1^ but a secondary optimum was found between 6.7 and 7.4 m s^−1^. For the Ivory Coast, the decrease was generally gradual from low to high wind speeds, while in Gabon the decrease was sharper for speeds above 6.2 m s^−1^. For both Ghana and Cameroon, there was a negative correlation between IChla and wind speed up to speeds of 6–6.5 m s^−1^ and a partial reversal for larger values. Wind speed appeared optimum in the range 3.9–5.3 m s^−1^ in the Nigeria system.

SLA was selected among the four most important predictors in seven systems. It was the second most important variable for Ghana (19%) and Nigeria (16%). For the other areas, the relative influence varied between 11% (Sierra Leone) and 18% (Gabon). SLA generally had a more modest influence on IChla than SST but it is worth observing that both quantities were well correlated in half of the cells (Fig. 3). The relationship between IChla and SLA was fairly diverse. An intermediate optimal range was found for three systems, Sierra Leone, Liberia and Ivory Coast over the intervals from −0.032 to 0.082 m, −0.028 to 0.051 m, and −0.024 to 0.046 m, respectively. Only in Ghana was a clear negative relationship between SLA and IChla observed. In contrast, the systems of Nigeria, Cameroon and Gabon were characterized by a general positive relationship between SLA and IChla.

EKE appeared among the four most important predictors in four systems, being the most important in Gabon (22% of relative influence) and the second one in Ivory Coast (12%). Its relative influence was otherwise in the interval 8% (Guinea-Bissau) to 15% (Liberia). In Guinea-Bissau, Liberia and Gabon systems, a negative correlation with IChla was observed, while the opposite was found for the Ivory Coast (with a plateau for EKE above 0.18 m^2^ s^−2^).

### 3.4 Regional results

In this section, the relationships between IChla and physical variables are discussed in more details by focusing on the annual cycles displayed in each system for the most influential variables. The two quantities yielding IChla were usually well correlated but some exceptions occurred, so that the mean distance from the coast to the eutrophic threshold (1 mg m^−3^ isoline) and the average Chla in the eutrophic area were illustrated together with IChla in Appendix 2. Climatological maps are also shown for selected variables in Appendices 3 and 4.

#### 3.4.1 Guinea–Bissau

The Guinea–Bissau system expands from cells 1 to 5 (see numbering of the cells in Fig. 1) covering the whole coastal area of Guinea–Bissau and part of Guinea. This system was characterized by having the widest continental shelf in the Gulf of Guinea, 172 km on average (Fig. 5A). It was also a system influenced by the Senegal Upwelling, characteristics reflected in an upwelling season in boreal winter months (Fig. 5C and D). In general, this area appeared as the continuity of the northwest African eastern boundary upwelling system (Carr and Kearns, 2003, Chavez and Messier, 2009).

The BRT model for this system accounted for 75% of the IChla variability (as expressed by $D^{2}$, Fig. 6). Even if SST, SLA and Front Index were highly correlated (|r| usually above 0.78, Fig. 3), the BRT model selected the Front Index (relative influence of 44%) and SST (21%) as the most significant variables, as seen in the previous section (Fig. 6).

**Fig. 8:**
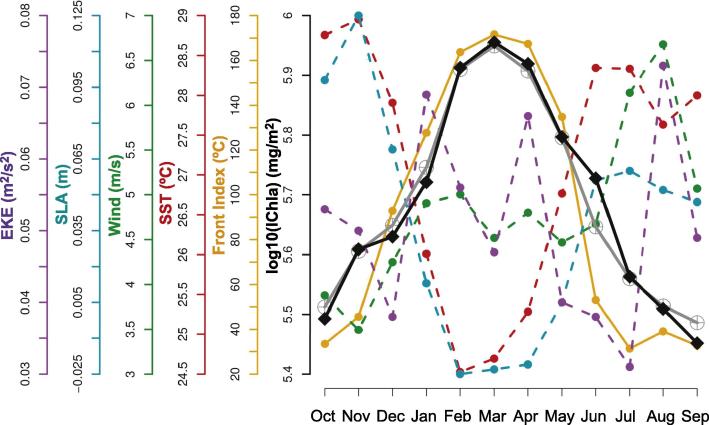
Guinea-Bissau system: Climatological monthly mean of IChla (black line), BRT-model fitted IChla (gray line) and the most significant physical variables according to the BRT results.

The highest IChla occurred between February and April when the Front Index was strongest, and the SST and SLA were lowest (Fig. 8). The average computed over the cells 1–5 for the period February-April was 24.8 °C and −2 cm for SST and SLA, respectively. In these months the wind speed was relatively moderate (average of 4.8 m s^−1^) compared with the rest of the year but the probability to observe upwelling-favorable northwest winds was maximum in the months where IChla was maximum (Fig. 5H). The eddy activity was more or less permanent during the whole year (Fig. 5F and G); consequently it could have a positive influence entraining water from the coast to offshore in the months when upwelling conditions were present but it seemed that eddies by themselves were not enough to maintain the highest IChla levels outside the upwelling season, and they were not selected as a major explanatory variable (Fig. 7). This being said, the IChla values remained fairly high yearlong if compared with the other sub-systems (Fig. 5B, Appendix 3). The mesoscale activity was consistent with high levels of EKE during the annual cycle (mostly for cells 3–5, Fig. 5I). It can be observed that the mean geostrophic flow remained low (Fig. 5J) in spite of high EKE. An examination of actual maps showed that large current amplitudes were found in association with eddies but they tended to cancel each other at the level of a cell (a phenomenon further compounded by temporal averaging).

The lowest IChla values were registered during August-October in association with the increase in SST and SLA (Fig. 8), with the average computed over the system for this period being 28.1 °C and 7 cm, respectively. The wind speed amplitude reached the maximum of the whole year in August (6.7 m s^−1^) but the direction was no longer favorable to upwelling, with a probability to observe winds from the northwest being close to zero in July-August (Fig. 5H). This probability increased slightly in September-October but wind speed was then lower.

#### 3.4.2 Sierra Leone

**Fig. 9:**
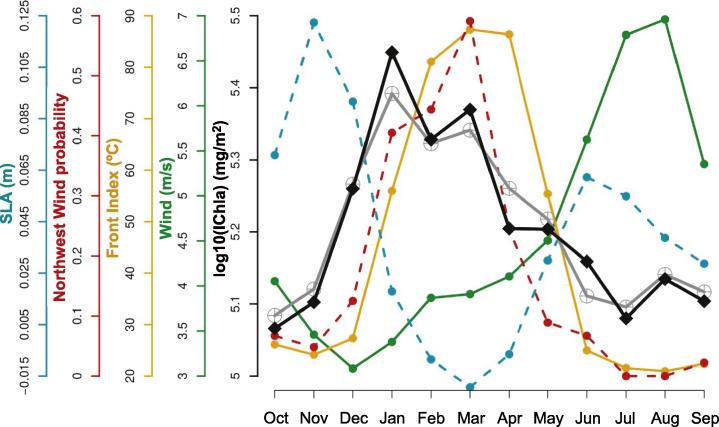
Sierra-Leone system: Climatological monthly mean of IChla (black line), BRT-model fitted IChla (gray line) and the most significant physical variables according to the BRT results.

The Guinea–Sierra Leone system corresponds to cells 6–10 covering the southern half of the coasts of Guinea and the northern half of Sierra Leone down to Sherbro Island (Fig. 1). The BRT model for this system accounted for 70% of the IChla variability. The variables related to wind were the most significant, accounting for a total of 36% of the relative influence (Fig. 6): 21% for wind speed and 15% for wind direction as discussed in Section 3.3. As in the previous system, the highest values of IChla were observed in boreal winter months (January-April, Fig. 9) when wind speed showed moderate values (coherently with the partial dependency, Fig. 7), on average 3.8 m s^−1^ over the cells 6–10 for this period, while the probability of northwestern upwelling-favorable winds was maximum (Figs. 5H and 9). On the other hand, as analyzed by the BRT model (Fig. 7), high wind speeds from the southwest (mostly found in summer) were not conducive to high IChla.

Similarly to Guinea-Bissau, the Front Index also had a large influence in the BRT model (20%), as had SST and EKE to a lower extent (10%), while SLA had an influence of 11% (higher than in the Guinea–Bissau system). Cyclonic and anticyclonic eddies had an influence of 6% and 8%, respectively. It can be noted that high numbers of eddies were only found in the western half of the system where the continental shelf was still wide (Fig. 5A, F, and G). But the division between systems is still justified by the sharp gradient between this area and the neighboring systems seen for other variables such as IChla, SST, SLA, EKE, the width of the shelf, and the respective influence of the independent variables in the model (Appendix 1).

This system shared some characteristics associated with the Guinea–Bissau upwelling area, like high values of IChla during the coldest months with strong fronts and negative values of SLA (Fig. 9), and fairly high correlations between SLA, SST and Front Index (Fig. 3), but there were also some differences. The increase in IChla anticipated by approximately one month the increase in frontal activity and the decrease in SST and SLA; this early increase could be related to a small increase in the eastward geostrophic current observed in December (Fig. 5J). There was also a slight increase of IChla observed in August (Fig. 9) that coincided with a second period of low SST (actually the minimum for the year). The IChla peak in August was more due to an increase of the average Chla in the coastal region than to an increase in the distance from the coast to the 1 mg m^−3^ isoline (Appendix 2). The flow from the Konkoure River with an outlet in Guinea (Fig. 2) and peak values in August could also concur to this higher Chla. During that period, there was an increase in EKE and in the number of cyclonic eddies that could be responsible for the extension of Chla distributions offshore.

#### 3.4.3 Liberia

**Fig. 10:**
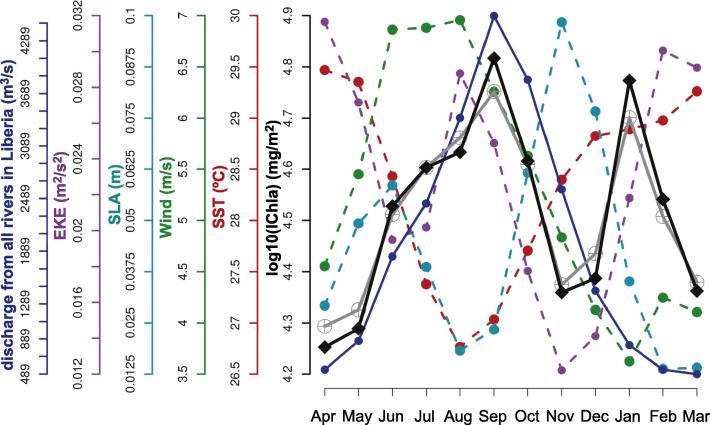
Liberia system: Climatological monthly mean of IChla (black line), BRT-model fitted IChla (gray line) and the most significant physical variables according to the BRT results. The sum of river discharge from all rivers in Liberia is based on published data.

The BRT model for the Liberia system (cells 11–21) showed a *D*^2^ of 74%, with the most significant variables being SST (20%), and wind speed (19%). Two peaks in IChla were observed in the average annual cycle (Fig. 10). Even though of similar amplitude, their duration differed, with an isolated peak in January, and a more extended peak from June to October. The IChla maximum in January was clearest in the western part of the area, coinciding with weak but predominantly northwest (upwelling-favorable) winds in the western cells, and could be associated with a similar pattern in boreal winter in the Sierra Leone system. Still in the western cells of the Liberia system, in winter SLA was lower and the Front Index and EKE higher (but with delayed peaks with respect to IChla, in February-March, Fig. 5D, E, and I). The IChla peak in January was mainly due to the distance from shore of the 1 mg m^−3^ isoline that reached the annual maximum in that month (42 km), and only secondarily to a slight increase in the average Chla (Appendix 2).

In contrast, the second peak of IChla in September was more representative of the regime observed within the systems further east in the GoG, with the major upwelling season observed in boreal summer. In this period, minima in SST and SLA were observed, coinciding with a fairly high Front Index and dominant winds from the south and southwest. Moreover the flow from the various tributaries feeding the Liberia coast (Moa, Mano, Lofa, Saint-Paul, Saint-John rivers) showed a substantial total (Fig. 2), with climatological maxima usually in the period August-October and a peak in September, in phase with IChla (Fig. 10). Another difference with the January peak was the dominance of higher wind speeds. A characteristic of the system was the (negative) correlation between SST and wind speed (around −0.62, Fig. 3) which was not found in neighboring systems. As seen in the partial dependency plots (Fig. 7), two ranges of wind speed were associated with high IChla, one of moderate values (<4.2 m s^−1^) and another of higher values (6.7–7.4 m s^−1^). This last optimal wind speed range was found mainly during May-September, with the maximum probability in July-August. For this region the wind direction parallel to the coast (favorable to upwelling) is from the northwest, but this direction was only found in boreal winter (mainly January-February). During the period May-October, the dominant direction was from the south/southwest, a direction more related to the Ivory Coast regime. The Liberia system thus appeared as a transition zone between the northwest Africa upwelling system and the upwelling centers found along the coasts of Ivory Coast and Ghana.

#### 3.4.4 Ivory Coast and Ghana

The marine regions of Ivory Coast and Ghana are the most documented along the Gulf of Guinea and have usually been regarded as a unique system within the region, because they have a continuous upwelling area and comparable seasonality (Djagoua et al., 2011, Djakoure et al., 2014, Wiafe and Nyadjro, 2015). The system of Ghana also comprises the coasts of Togo (Fig. 1). Although both areas are described in the same section, they were split in two different systems based on the BRT results and to allow differences to be distinguished (Appendix 1). The BRT model for the Ivory Coast (cells 22–30) and Ghana (cells 31–37) systems yielded a deviance value $D^{2}$ equal to 91% and 76%, respectively.

**Fig. 11:**
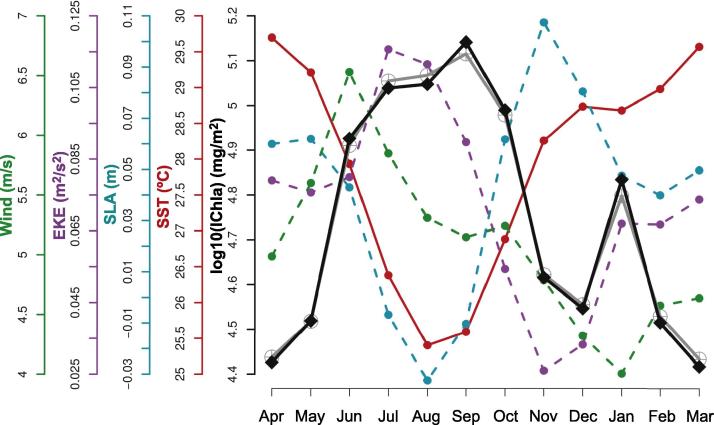
Ivory-Coast system: Climatological monthly mean of IChla (black line), BRT-model fitted IChla (gray line) and the most significant physical variables according to the BRT results.

**Fig. 12:**
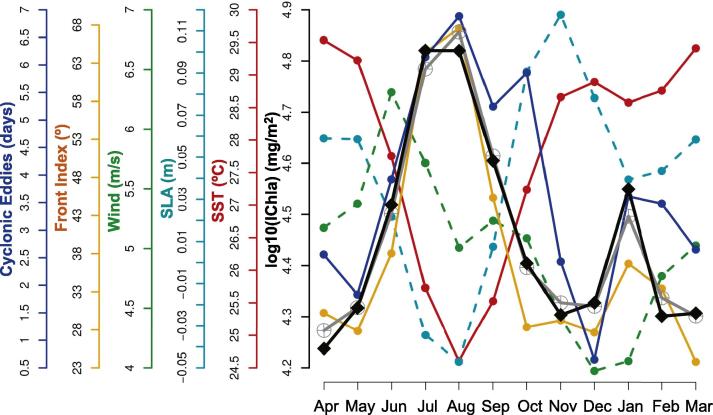
Ghana system: Climatological monthly mean of IChla (black line), BRT-model fitted IChla (gray line) and the most significant physical variables according to the BRT results.

Even if the relative importance of the physical variables on IChla variability showed similar patterns (Fig. 6) and SST was the most influential variable in both regions, SST had a higher relative influence (46%) in the Ivory Coast region compared with Ghana (32%). A similar percentage (9–12%) of relative influence for SLA, EKE, wind and Front Index was observed in the Ivory Coast. The second most significant variable in Ghana was SLA with 19% of relative influence, which was actually the highest score for SLA in the whole GoG domain. For the Ivory Coast and Ghana regions, the existence of two upwelling periods has been documented: a major upwelling in boreal summer and a minor upwelling peaking in January (Arfi et al., 1991). Actually, the main (summer) upwelling period extended from June to October, particularly for the Ivory Coast system (Fig. 11, Fig. 12). It was clearly associated with the characteristics of upwelling events: cold SST, negative SLA, and frontal activity (Fig. 5C, D, E, Appendix 4). During this period winds were predominantly from the south/southwest, meaning that there was an along-shore (upwelling-favorable) component where the coast curved to the north, just east of the Capes Palmas and Three-Points (Fig. 1). For the minor (winter) upwelling, these characteristics were not observed, or to a much lesser extent: SST was just slightly colder than during the warm season, SLA showed only a secondary minimum, and the Front Index had a secondary maximum in February along the Ivory Coast (Fig. 5E) and in January in Ghana (Fig. 12). Addison (2010) pointed out that the minor upwelling has a duration of around three weeks, suggesting that the variations of the associated physical characteristics might be only partly picked up by the monthly series used here. In general, the upwelling seasons were associated with an increased Front Index, but it was higher in the Ivory Coast system with respect to the Ghana area (Fig. 5E). The maximum Front Index observed for the Ivory Coast was found fairly late in the summer upwelling season, in August. As in the Guinea–Bissau system, SST, SLA and Front Index were well correlated (Fig. 3). It is also worth noticing that both Ivory Coast and Ghana were characterized by a narrow continental shelf (Fig. 5A), with the exception of an extension around cell 32 in the Ghana area; this might actually be relevant considering that it happens to be the location of the maximum IChla associated with the summer upwelling (Fig. 5B). Finally, the influence of rivers on IChla was likely limited at the scale of the overall region even though it could have a local impact (Kouadio et al., 2013). The four major rivers of Ivory Coast (Cavally, Sassandra, Bandama and Comoé) have a fairly small flow (Fig. 2) usually peaking in October (Kouadio et al., 2013) when IChla was observed starting its decrease, while the climatological maximum of the Volta River outflow in October coincided with a low IChla in the Ghana system.

The Ivory Coast/Ghana upwelling occurs along a mostly zonally-oriented coast, a topography at variance with the main eastern boundary upwelling systems where the coast is oriented in a meridional direction. As said above, the summer upwelling otherwise shares common characteristics with its eastern boundary counterparts, with a negative SLA signal associated with a shoaling of the thermocline and nutricline (Houghton, 1976, Verstraete, 1992, Colin et al., 1993, Herbland and Loeuff, 1993). The origins of the upwelling processes occurring in the region has been discussed for a long time with several possible mechanisms summarized by Roy (1995).

With respect to eastern boundary upwelling centers, the role of wind in the generation of upwelling has been debated because of a lack of correspondence (in space and/or time) between the two quantities (Houghton, 1976, Bakun, 1978, Verstraete et al., 1980). This is in line with the fairly limited seasonal variations of the wind field in the Ivory Coast and Ghana systems (Fig. 5H). If upwelling conditions are linked to a favorable environment for phytoplankton growth, this is also coherent with the result of the BRT model showing that wind speed played a relatively small part in the IChla variability (11% and 13% for Ivory Coast and Ghana, respectively, Fig. 7). A more recent study by Wiafe and Nyadjro (2015) showed that Ekman transport was strongly conducive to upwelling in summer in the eastern part of the Ghana system only (east of 1°W, cells 34–39), while Ekman pumping was usually not responsible for significant upwelling. Consistent with the increase in offshore Ekman transport in the eastern part of the Ghana system was the increase of the along-shore wind in summer (Fig. 5H). Another explanation for the summer upwelling could be the acceleration of the Guinea Current in this season (Fig. 5J), whereby the thermocline would be further tilted towards the coast by geostrophic balance (Ingham, 1970), but the importance of this phenomenon has been questioned by numerical tests (Philander, 1979). This increased current speed (magnitude >0.5 m s^−1^) was prominent in the Ivory Coast system in summer (in association with an increase in EKE, Fig. 5I) in phase with the indicators of upwelling and with IChla, but the Guinea Current was also very energetic in spring when IChla was at a minimum. As far as the Ghana system is concerned, the average current was fairly strong (albeit less than in the Ivory Coast) only in the western-most cells and it was rather constant during the year (Fig. 5J) even though EKE peaked during the summer upwelling in the west. This being said, Colin (1991) still emphasized the role of wind and Guinea Current, while Jouanno et al. (2011) related the summer cooling to vertical mixing induced by the modulation of velocity shear at the base of the Guinea Current.

The role of capes has also been proposed whereby cyclonic eddies are generated downstream of the Capes Palmas and Three-Points by their interaction with the Guinea Current (Marchal and Picaut, 1977). A high number of cyclonic eddies were indeed seen in summer for the Ivory Coast system and to a lower extent in the Ghana system, while anticyclonic eddies were also detected along the Ivory Coast earlier in the season, in May-July (Figs. 5F, G and 12). On the other hand, anticyclonic eddies were less observed in the Ghana system. A recent numerical analysis by Djakoure et al. (2014) identified about two cyclonic eddies per year in the lee of the capes, which was consistent with the location of cyclonic eddies in cells 23–25 east of Cape Palmas, and in the central part of the Ghana system. It is stressed here that the present study identified all eddies, not only those associated with the interactions with the capes; furthermore, the eddy field was quantified as a number of days when eddies were detected within a cell so that this number was not directly comparable with those of Djakoure et al. (2014). As for the effect on upwelling, based on a numerical analysis, Djakoure et al. (2014) suggested that the generation of cyclonic eddies associated with the capes played little role in the upwelling. This is consistent with the fact that the eddy field explained little of the IChla variability according to the BRT model (Fig. 6), notwithstanding the remarkable correspondence between the number of cyclonic eddies and IChla for the cells 23–25 from July to October. On the other hand, this correspondence was not verified around March, when the cyclonic eddy activity was again high but IChla was at a minimum at the start of the warm season (Fig. 5B and F). Even though eddies might not be the ultimate driver of the IChla variations, they likely played a role as energetic structures distributing Chla across the domain.

The hypothesis of a remote forcing for the generation of upwelling in the considered systems has also been discussed (Moore et al., 1978, Adamec and O’Brien, 1978, Servain et al., 1982, Picaut, 1983). In that scheme, the intensification of the easterlies in the western Equatorial Atlantic in spring generates an upwelling Kelvin wave traveling eastward; upon reaching the African coast, this splits into coastally trapped Kelvin waves propagating poleward and then westward in the case of the northern shore of the Gulf of Guinea. This hypothesis was questioned by Colin et al. (1993) on the basis of wave phasing and considering that the offshore extension of the thermocline oscillation was found largely exceeding the Rossby radius. It was however revisited using satellite data by Polo et al. (2008) and Wiafe and Nyadjro (2015). To further explore this issue with the data presented here was out of the scope of this study, but the discussion highlighted the complexity of the dynamics taking place in the region and how their interactions could change in space and time.

#### 3.4.5 Nigeria

**Fig. 13:**
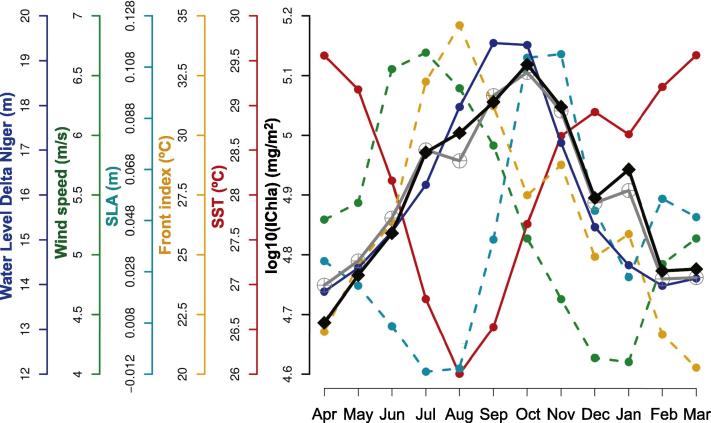
Nigeria system: Climatological monthly mean of IChla (black line), BRT-model fitted IChla (gray line) and the most significant physical variables according to the BRT results. The water level for the Niger Delta is from altimetry data (see text).

The Nigeria system (cells 38–47) was characterized by a wider continental shelf (51 km) with respect to the Liberia (37 km), Ivory Coast (31 km) and Ghana (46 km) systems (Fig. 5A). The relative influence of the predictor variables was fairly evenly distributed compared to other systems (Fig. 6), with the most influential being SST (27%), SLA (16%) and wind speed (15%). The average IChla over the system showed a broad period of relatively high values from June to February (Fig. 13) but the variations of IChla displayed a certain diversity within the system: there was a clear peak in October in the western part (centered on cell 42), and a period of high values from September to January in the eastern part (Fig. 5B).

IChla started increasing in April and was already high in July-August, when the system displayed some characteristics associated with upwelling, cold SST between July and September (with the minimum for the whole year in August), minimum negative SLA during July-August, a relatively high Front Index, and sustained winds (Fig. 13). Ibe and Ajayi (1985) pointed the existence, at least in certain years, of a wind-related upwelling between July and September but the recent climatology did not suggest the dominance of winds favorable to upwelling (Fig. 5H). As in the Liberia system, SST and wind appeared fairly well (negatively) correlated (Fig. 3). IChla remained high from September to December (peak in October), while upwelling conditions receded (increasing SST and SLA, with the peak SLA occurring in October-November).

**Fig. 14:**
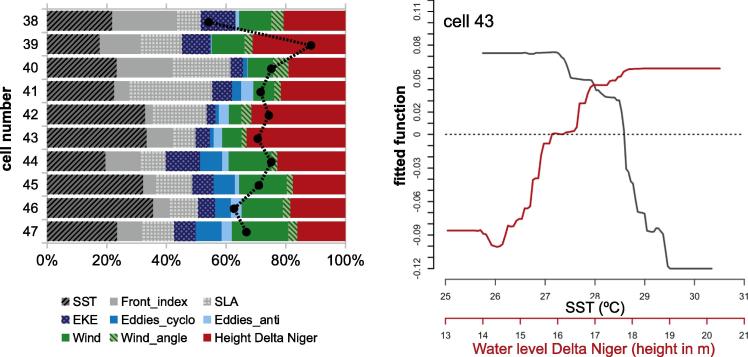
(A) BRT results by cell of relative influence of physical variables on IChla for the Nigeria system. (B) Partial dependence plots for the water level of Niger River and SST in the BRT model for cell 43.

It appeared that the physical variables considered so far were insufficient to fully explain the IChla variability in the Nigeria system; this was consistent with the fact that the BRT model’s deviance amounted to 54%, the minimum found among the GoG systems (Fig. 6). It then seemed worth exploring the influence of the Niger River discharge in the system, as represented by satellite-derived water level at downstream stations (see Section 2.6). To account for the fact that the river discharge was a local phenomenon, the BRT analysis was repeated adding the water level as predictor variable for each cell in the Nigeria system. The BRT models for the cells 38–47 yielded a percent of deviance explained $D^{2}$ between 54% and 88% (mean 71%), with the most influential variables being SST and Niger water level (average relative influence for cells 38 to 47 of 26% and 23%, respectively, Fig. 14). The maximum influence of the water level was found in the cells 42–43 (approximately 33%). From the plot of partial dependency (Fig. 14B), IChla appeared inversely related to SST (as already noted if the water level was not included in the predictor variables, Fig. 7), and well correlated with water level. The latter point also appeared clearly comparing the similar seasonal cycle shown by IChla and the water level that peaked in September-October (Fig. 13; $R^{2}$ of 0.81).

For completeness it is noted that a small peak in EKE and the number of anticyclonic eddies was observed in October. There was also a peak in April (when actually the number of cyclonic eddies reached its maximum, Fig. 5F) but this corresponded to the minimum IChla. These results suggested that eddies were not a major factor in the IChla increase but could still play a role in the offshore transport of Chla during the months of maximum influence of the river discharge. An isolated peak of IChla was also registered in January. Ibe and Ajayi (1985) mentioned a possible minor upwelling along the Nigerian coast between December and February but their study did not find evidence of this phenomenon. Fig. 13 showed a slight decrease of SST and SLA in January that could be an indicator of a minor upwelling during this month.

#### 3.4.6 Cameroon

The Cameroon system (cells 48–52) was characterized by a more meridional coast compared with the countries westward and by the presence near the coast of the Bioko Island (Fig. 1). The continental shelf is largest in the cells associated with Bioko Island (cells 49–50) where IChla appeared the largest on average (Fig. 5B). The value of $D^{2}$ obtained by BRT modeling was 59%, the most influential variables being SST (24%), SLA (16%) and Front Index (16%). Differently from the systems that were characterized by upwelling phenomena such as Guinea-Bissau, Ivory Coast and Ghana, SST, Front Index and SLA were not correlated, but SST appeared moderately (negatively) correlated with EKE and wind (as in the Nigeria system, Fig. 3).

**Fig. 15:**
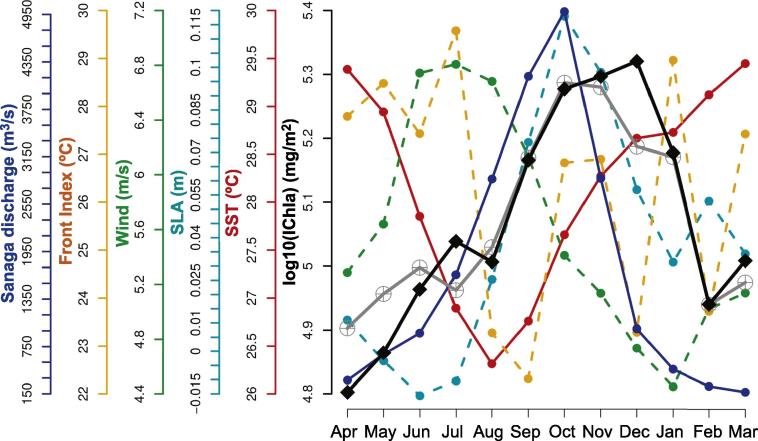
Cameroon system: Climatological monthly mean of IChla (black line), BRT-model fitted IChla (gray line) and the most significant physical variables according to the BRT results. The Sanaga River discharge is based on published data.

IChla started increasing in June-July when SST was decreasing and SLA was at a minimum, but the broad IChla maximum (September-January) was associated with high values of SLA and SST (actually, SST and IChla increased in parallel from August to December, Fig. 15). There existed a cyclonic eddy activity in this system with a maximum in March-May (concurrent with a fairly high Front Index), particularly in cells 50–51 close to Bioko Island. Even though again eddies could be important to distribute phytoplankton across the domain, the number of eddies appeared as a weakly influential predictor (6%) and the maximum occurrence was coincident with the minimum IChla. As for the Nigeria system, the influence of local freshwater flow was considered with the Sanaga River being the most important tributary in terms of discharge in the system (Fig. 2). IChla increased with its flow from April to October but was still high after the drop in discharge (Fig. 15). Unfortunately, we were not able to find monthly data covering the period of study so that only climatological data could be presented and the river discharge could not be included in a BRT analysis. The Bight of Biafra (containing the coast of Cameroon) is also the area within the GoG and adjacent lands receiving the largest precipitations (which can result in land run-off), and there is a significant amount of freshwater that enters the system across the whole year (Berger et al., 2014).

River discharge and the presence of the Bioko Island are additional factors of complexity in that system, which is otherwise ecologically rich. For instance, the maximum of nesting activity of sea turtles in the ecologically important community of Bioko Island is in December-January (Tomás et al., 2010), coincident with the peak in IChla in cells 49–51. So progress is still needed for an improved understanding of the phytoplankton variability in the region, its dependence on the physical environment, and how it can be related to higher trophic levels.

#### 3.4.7 Gabon

The Gabon system extended from cells 53 to 56 covering the southern half of Equatorial Guinea and the northern part of Gabon (until Cape Lopez). After Ivory Coast, it had the second smallest width of continental shelf (32 km). The percentage of deviance explained by the BRT model was 60%, with a fairly even distribution of relative influence among predictors (Fig. 6), from 16% (for SLA) to 22% (EKE), except for wind direction (4%). For this system the number of cyclonic and anticyclonic eddies were not included among the predictors because these structures were almost absent in the region (Fig. 5F and G).

**Fig. 16:**
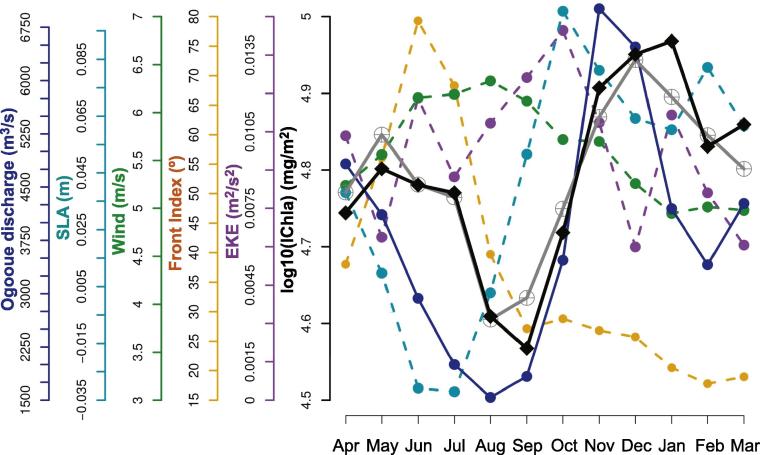
Gabon system: Climatological monthly mean of IChla (black line), BRT-model fitted IChla (gray line) and the most significant physical variables according to the BRT results. The Ogooue River discharge is based on published data.

The IChla maximum was observed in boreal winter (November-January) and was due mostly to the extension offshore of the 1 mg m^−3^ isoline (Appendix 2). The minimum occurred in summer (August-September) in association with low SST and SLA (Figs. 5C, D and 16). As for the previous two systems, river flow appeared as a possible influence on the IChla variability. The Ogooue River is located out of the system, slightly south of the southern-most cell (56) considered here, but it represents the second largest discharge in the GoG (after the Niger, Fig. 2). Overall, the Ogooue River discharge and IChla appeared well correlated but IChla remained high (at its maximum value) after the flow started to drop in January (Mahé et al., 2013), a phenomenon repeated for a secondary peak in March-May (Fig. 16). Besides the Ogooue, it was not excluded that other rivers might have an impact on the Gabon system, including the Congo River located far south: its maximum flow is observed in November-January with a plume extending hundreds of km and even beyond Cape Lopez in boreal winter (Denamiel et al., 2013, Hopkins et al., 2013), but some studies suggest that its influence is limited there (Eisma and van Bennekom, 1978).

The Front Index was often well (positively) correlated with IChla (particularly in regions with upwelling characteristics such as Guinea–Bissau, Ivory Coast, Ghana), but this was not the case in the Gabon system. The Front Index showed a distinct maximum in boreal spring and summer (Fig. 16) when SST and SLA were lowest. Located near the Equator, this system is highly influenced by the equatorial dynamics. The latitudinal oceanic gradient of SST had a marked influence on the Front Index values between April and August (Appendix 4) due to the difference between warm temperatures north of the Equator and cold ones further south. The high Front Index values were not associated with local, coastal, dynamics but were part of the general equatorial dynamics: Front Index, SST and SLA in the Gabon system were inter-related (particularly in the southern-most cells, Fig. 3) and were strongly related to values offshore of the system (Appendix 4), with $R^{2}$ of 0.99, 0.92 and 0.99 respectively when comparing the average between 0–200 km and 200–400 km. Therefore this system is to be seen as the southern-most boundary area of the GoG.

## 4 Conclusions

The Gulf of Guinea includes a wide tract of African coast, covering more than 25 degrees of longitude over a restricted latitudinal range (13 degrees). It is a diverse and productive system but it is also under pressure from climate change and anthropogenic influences so that it is important to understand the variations in phytoplankton communities in its coastal regions, and particularly how they respond to physical forcing within their environment. This study contributed to that effort by revisiting the seasonal cycles of Chla and relevant physical variables along its coasts and analyzing their relationships through a statistical approach (BRT modeling) applied to observational data. This analysis appeared feasible with the availability of a decade or more of satellite products describing the major physical variables of potential interest to understand Chla variability. Furthermore, the use of OC_CCI Chla products enabled sufficient distribution on monthly scale in space (across the domain) and in time (for all seasons) (Fig. 4). So far the very poor coverage afforded by other ocean color products in certain parts of the annual cycle and particularly in certain regions limited our ability to conduct a thorough and balanced analysis of the Chla variations. Physical variables were free from the effects of cloud coverage, being based on merged microwave-infrared data (SST), altimetry (SLA, EKE) and scatterometer (winds).

BRT modeling is a powerful statistical approach that is able to model complex non-linear relationships between predictor variables and the output variable while handling outliers and interactions between predictors. It was applied to an integrated value IChla representing the areal concentration within the eutrophic coastal zone, integrating the local variations in the amplitude of the Chla values and their extension offshore (Section 2.1). In all cases, the BRT models fitted the observed IChla well (Fig. 8, Fig. 9, Fig. 10, Fig. 11, Fig. 12, Fig. 13, Fig. 14, Fig. 15, Fig. 16). Even though they were not based on mechanistic relationships, the BRT results could shed some light on the forces driving the Chla variability, and certainly they could provide a view of the behavior between Chla and physical environment, and phytoplankton optimal environmental windows (Fig. 7), that should be expected from simulations of ecological models. The current study also highlighted some features that a successful ecological modeling should possess: besides a proper description of ecological interactions and physical fields (particularly the various upwelling phenomena), boundary conditions should be well represented in the northwest (in relation with the Northwest African upwelling) and the southeast (in relation to equatorial dynamics), and the river discharge should be accurately described. From the BRT analysis, it appeared that the relative influence of each physical predictor varied between systems (Fig. 6), except for eddy dynamics that did not seem to have a significant influence in any system; it could however be surmised that eddies played an important role in the distribution of Chla fields within each region.

One of the results of the study was a partition of the GoG domain into coherent systems (Table 1). This division was based on the results of the BRT model and observed variations associated with the various fields. It is however acknowledged that this partition was partly subjective and remains a difficult task to achieve: some variables showed clearly delineated features from one system to the next but other variables did not. Upwelling was a defining property of the GoG system, mostly through two different manifestations with their own seasonality: in the western-most part of the GoG (Guinea-Bissau, Sierra Leone and part of Liberia) that shared some characteristics with the broader Northwest African upwelling (Carr and Kearns, 2003), and the more independent Ivory Coast - Ghana system with a main upwelling season in boreal summer. In general, upwelling conditions with cold SST, negative SLA, fairly strong frontal activity, and moderate winds, appeared as the environmental window most favorable to high Chla values. Between the two ensembles of systems (Guinea-Bissau to Liberia, and Ivory Coast - Ghana), the differences were not only in terms of upwelling timing. To simplify, while the former still had the characteristics of Ekman-type upwelling regions, this was not the case of the latter. This might have significant ecological implications for higher trophic levels: in Ekman-type upwelling, recruitment might increase with upwelling intensity up to a point where turbulence (favored by increasing winds) becomes detrimental (dome-shaped relationship), whereas this relationship is more linear in the Ivory Coast - Ghana system (Cury and Roy, 1989).

The lowest percentage of deviance explained by the BRT model was found for the three eastern-most sub-systems (Fig. 6), when only oceanographic physical variables were employed, which raised the question of the potential impact from rivers. Because of issues related to data availability we were able to include river data in BRT modeling only in the case of the Niger River, which resulted in a significant increase of $D^{2}$ (Fig. 14). For the other systems, we resorted to monthly climatological discharge for the Sanaga and Ogooue rivers. In all three cases, it seemed that river discharge played a significant role in the regional variability of satellite derived Chla. Rivers can indeed provide nutrients and organic matter that can feed the coastal GoG and its phytoplankton communities (da Cunha and Buitenhuis, 2013, Arauja et al., 2014). It is also true that the use of the standard maximum-band-ratio algorithm OC4v6 (O’Reilly et al., 2000) in coastal, optically-complex, waters might lead to higher levels of uncertainties with respect to open ocean conditions. These uncertainties are often related to overestimated Chla values (e.g., Zhang et al., 2006, Mélin et al., 2007, Sá et al., 2015), as the algorithm does not properly handle excess amounts of inorganic particles (sediments) and chromophoric dissolved organic matter (CDOM) that can be associated with the river plume (Jourdin et al., 2006). Djagoua et al. (2011) and Kouadio et al. (2013) indicated that the influence of rivers was limited in the overall GoG or restricted locally to the estuaries, but the current study suggested that river discharge might have a significant impact around the Niger Delta and the Biafra Bight and that more work with appropriate bio-optical algorithms is required in these regions. More data on the estuaries feeding the GoG, including properties like nutrient concentrations or transparency, would be also very useful.

The present study presented an overview of the relationships existing between physical environment and Chla, highlighting the main features of the GoG as well as its complexity. It focused on the seasonal variations characterizing the GoG system but the observed relationships might tell us what possible evolutions are in store for phytoplankton as the physical environment is affected by climate change.

## Figures and Tables

**Table 1:** Vertices for each system constituting a partition of the Gulf of Guinea.
