# Supplementary material for: Variability of chlorophyll-a concentration in the Gulf of Guinea and its relation to physical oceanographic variables
Source: Prog Oceanogr. 2017 Feb;151:97–115. doi: 10.1016/j.pocean.2016.11.009 (PMC5339419; doi:10.1016/j.pocean.2016.11.009)
Supplement: Supplementary file 1 [file mmc1.pdf]

Supplementary Material

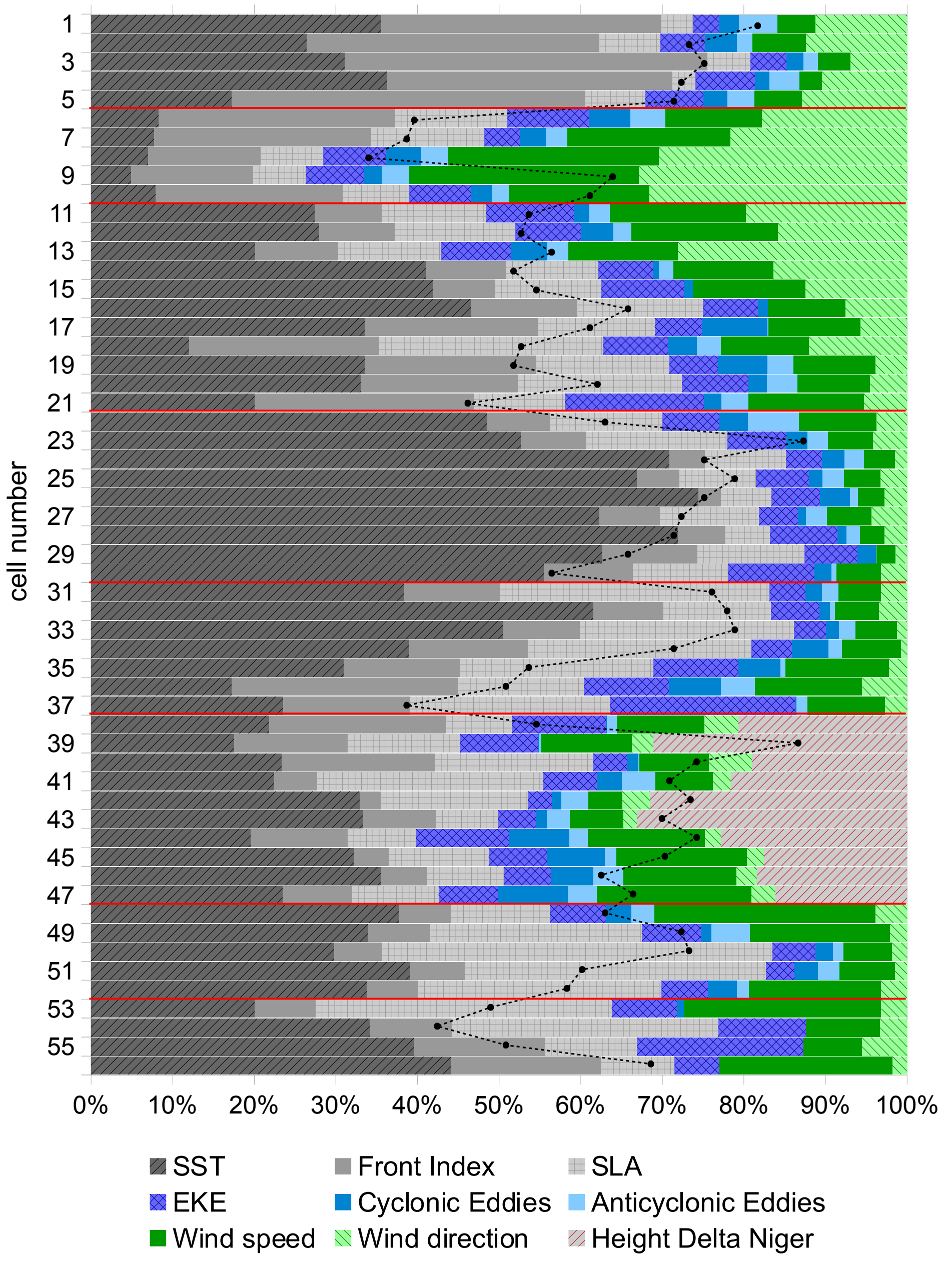

Appendix 1. Relative influence by cell (red lines show the partition into systems) along the Gulf of Guinea, and percentage of deviance explained D^2 (dashed black lines with dots).

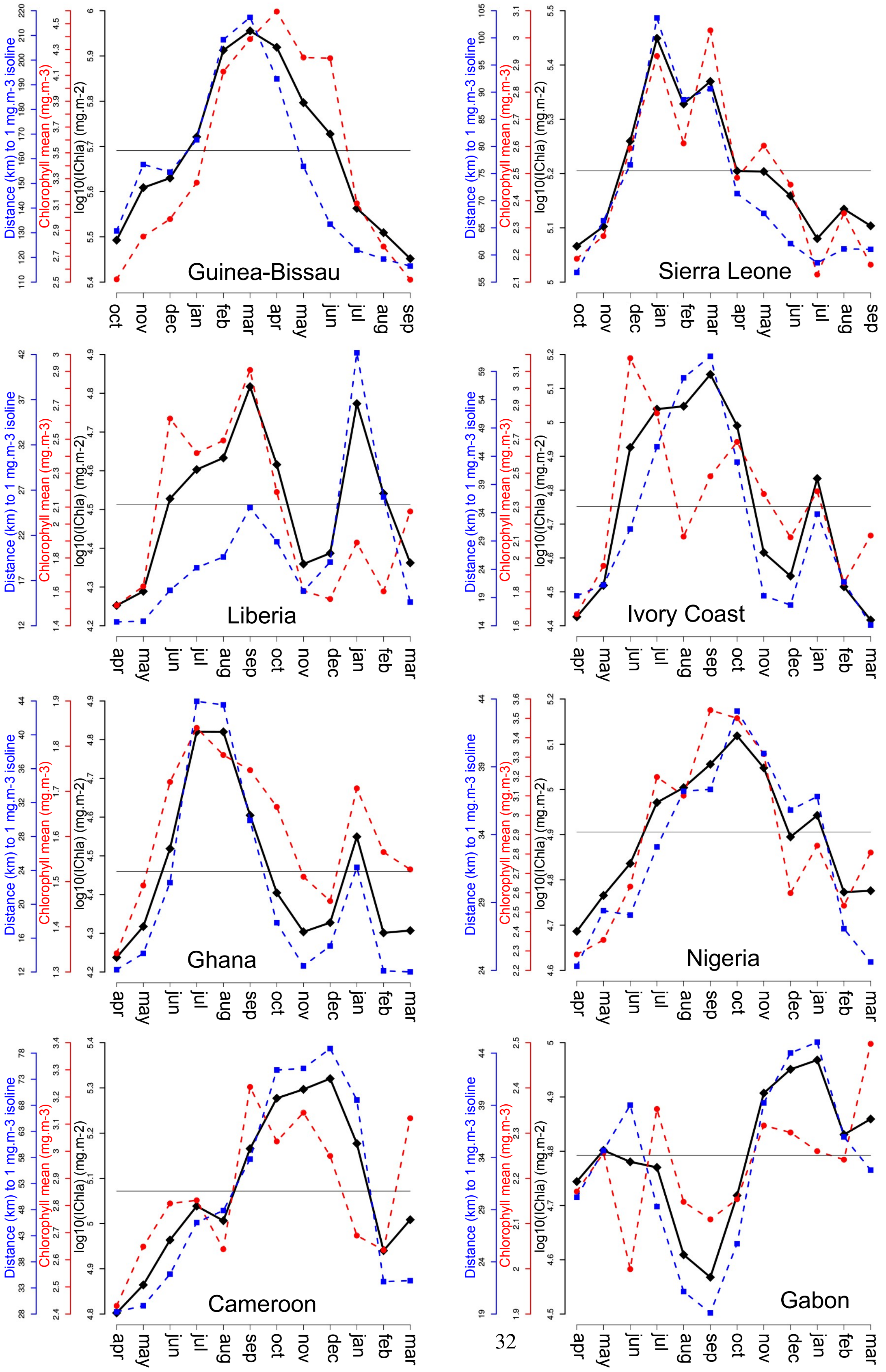

Appendix 2. For each system, monthly climatology of the distance from the coast to the 1 mg m^{-3} Chla isoline, the average Chla value within the eutrophic area (concentrations larger than 1 mg m^{-3}), and the product between these two quantities, IChla. The solid black line X-axis is the annual average IChla.

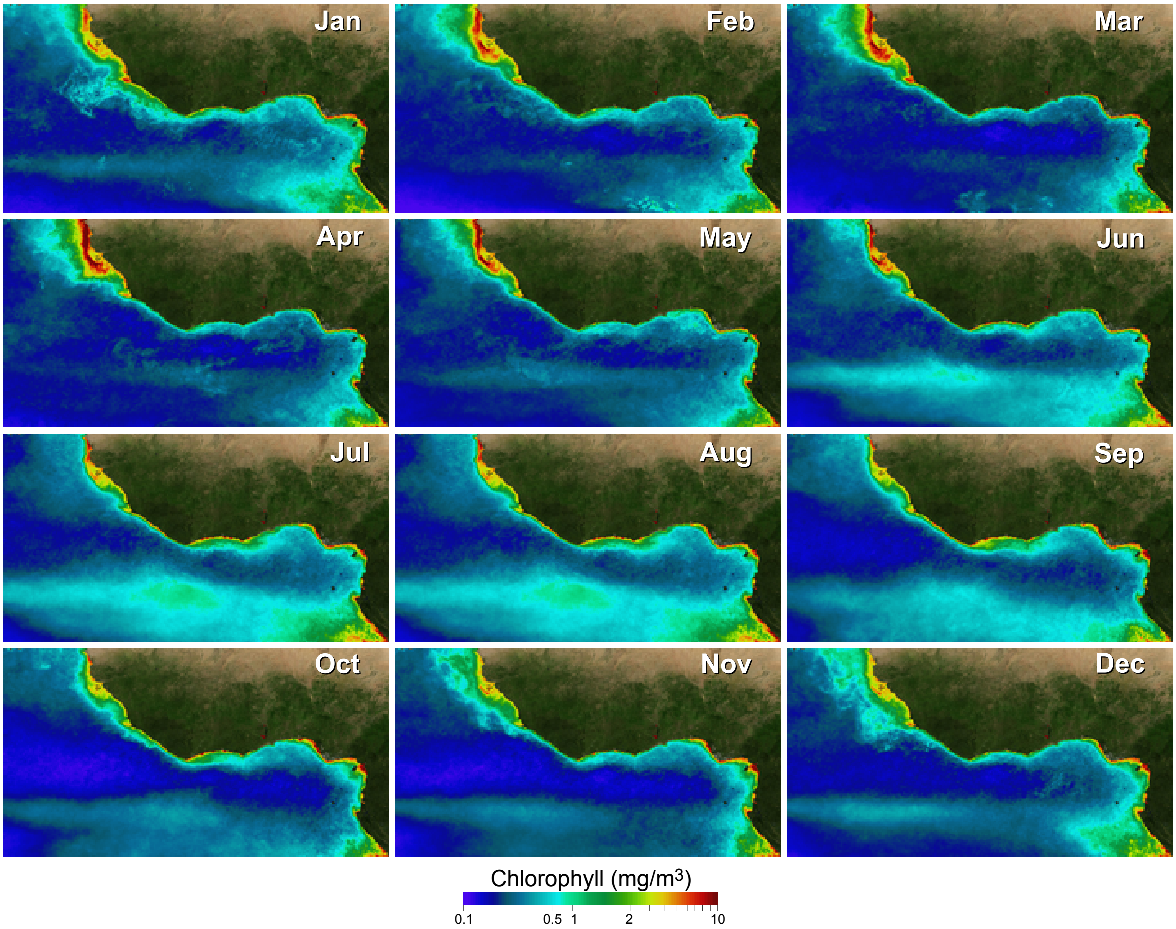

Appendix 3. Monthly chlorophyll-a climatological images (2002-2012) from OC_CCI over the Gulf of Guinea.

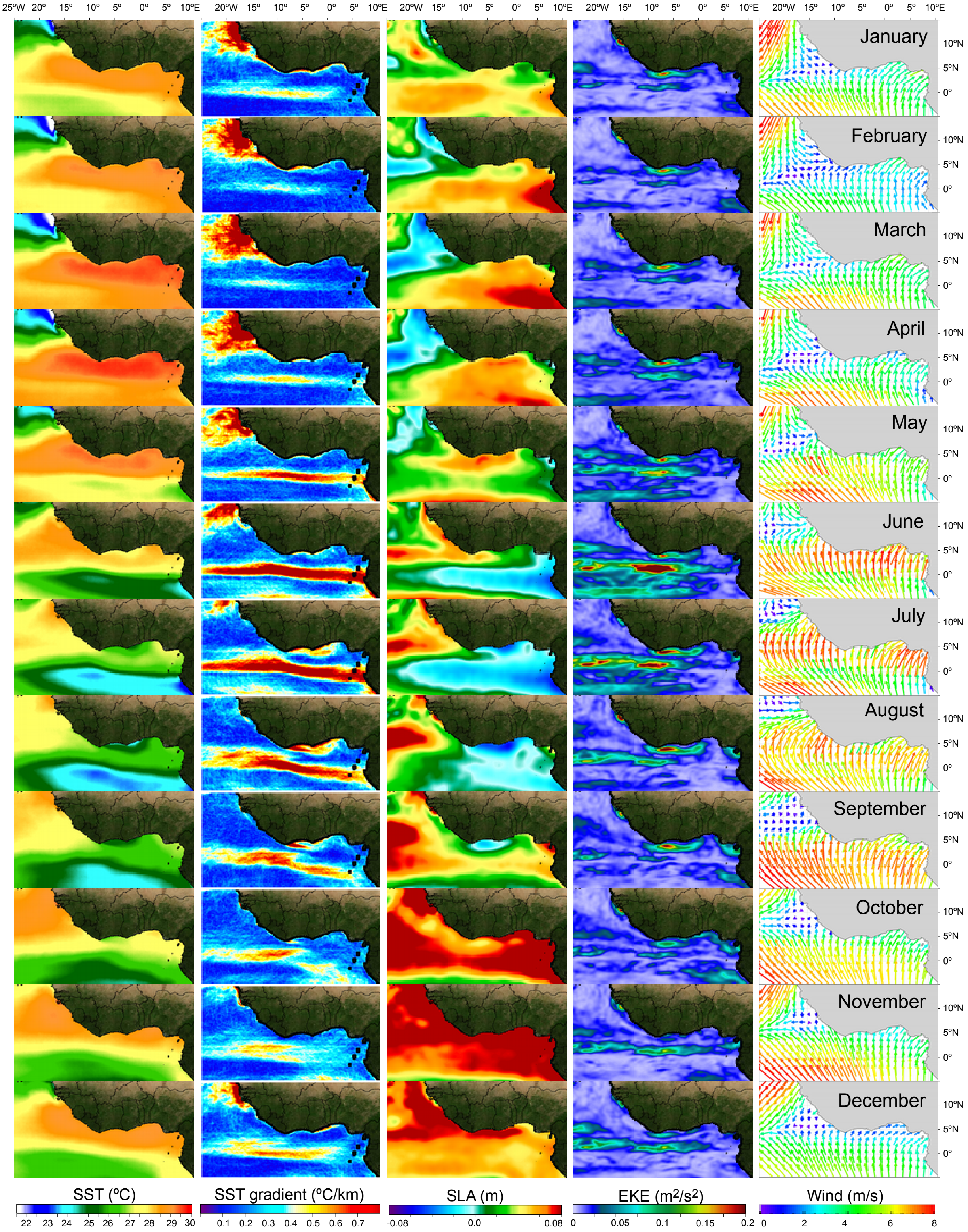

Appendix 4. Monthly climatological images (2002-2012) of physical variables over the Gulf of Guinea.
